# International pediatric surgery partnerships in sub-Saharan Africa: a scoping literature review

**DOI:** 10.1080/16549716.2022.2111780

**Published:** 2022-09-01

**Authors:** Alison Woods, Charles Shofner, Bethany Hodge

**Affiliations:** a Department of Pediatrics, University of Minnesota School of Medicine, Minneapolis, MN, USA; b Department of Pediatrics, Indiana University School of Medicine, Indianapolis, IN, USA; c Department of Pediatrics, Division of Pediatric Hospital Medicine, Global Education Office, University of Louisville School of Medicine, Louisville, KY, USA

**Keywords:** Global health, capacity building, education, academic medicine, sustainable

## Abstract

**Background:**

Sub-Saharan Africa (SSA) faces a critical shortage of pediatric surgical providers. International partnerships can play an important role in pediatric surgical capacity building but must be ethical and sustainable.

**Objective:**

The purpose of this study is to perform a scoping literature review of international pediatric surgery partnerships in SSA from 2009 to 2019. We aim to categorize and critically assess past partnerships to aid in future capacity-building efforts.

**Methods:**

We performed a scoping literature review following the Preferred Reporting Items for Systematic Reviews and Meta-Analyses for Scoping Reviews (PRISMA-ScR) guidelines. We searched the PubMed and Embase databases for articles published from 2009 to 2019 using 24 keywords. Articles were selected according to inclusion criteria and assessed by two readers. Descriptive analyses of the data collected were conducted in Excel.

**Results:**

A total of 2376 articles were identified. After duplicates were removed, 405 articles were screened. In total, 83 articles were assessed for eligibility, and 62 were included in the review. The most common partnership category was short-term surgical trip (28 articles, 45%). A total of 35 articles (56%) included education of host country providers as part of the partnership. Only 45% of partnerships included follow-up care, and 50% included postoperative outcomes when applicable.

**Conclusions:**

To increase sustainability, more partnerships must include education of local health-care providers, and short-term surgical trips must be integrated into long-term partnerships. More partnerships need to report postoperative outcomes and ensure follow-up care. Educating peri-operative providers, training general surgeons in common pediatric procedures, and increasing telehealth use are other goals for future partnerships.

## Background

An estimated five billion people worldwide do not have access to surgical and anesthesia care. The disparity in surgical care availability disproportionately affects sub-Saharan Africa (SSA), where an estimated 93% of the population do not have access [1]. When addressing the shortage of surgical care in SSA, specific consideration must be given to the pediatric population. According to UNICEF’s Generation 2030 Africa 2.0 report, 47% of Africa’s population is under 18 years old (yo), which accounts for 25% of the world’s children. Children currently represent more than half of the total population in one-third of the countries in Africa. By 2030, Africa’s under-18 population will increase by 170 million [2].

Most of the child population increase in the continent will occur in SSA. By the end of the century, births in Western and Eastern Africa will account for two-thirds of all births in the continent. The proportion of the continent’s births that occur in Northern Africa has decreased since 1950, with decline projected to continue [2].

To address the overall shortage of surgical providers in low-and-middle-income countries (LMICs), the Lancet Global Surgery 2030 report recommends that each country scale up their surgical workforce to 20 surgical providers per 100,000 population by 2030. To achieve this goal, approximately 1.27 million additional surgical providers will need to be trained [1]. When considering pediatric surgery, in the United States it is estimated that one pediatric surgeon is needed for every 100,000 children 0–15 yo, yet in Africa there is an estimated one pediatric surgeon for every six million children 0–14 yo [3].

For many years, global health initiatives have prioritized individual and communicable diseases, while relatively less attention has been given to scaling up surgical care in LMICs [1,4,5]. However, surgical conditions comprise 28–32% of the global disease burden. In 2010, more lives were lost worldwide from surgical conditions than from HIV/AIDS, tuberculosis, and malaria combined, and the non-communicable disease burden will continue to increase in coming years [1].

The Lancet Global Surgery 2030 report points out the important role that global health efforts, non-governmental organizations (NGOs), and other international partners can play in scaling up surgical capacity in LMICs, particularly regarding education and training [1]. Given the great disparity in surgical care in SSA and the current and future need for pediatric surgery in the region, increasing pediatric surgery capacity in SSA should be a global health priority.

In recent years, the strategy of global health efforts has shifted from short-term initiatives with little long-term impact towards more sustainable models that prioritize the needs of the local community [6]. The downfalls of short-term global health initiatives are particularly apparent in surgery, including a lack of follow-up care after visiting surgeons leave, variable long-term benefit, and a lack of accountability for poor postoperative (post-op) outcomes, among others [6–8]. Recommendations for ethical, sustainable global surgery efforts suggest that all initiatives have a training component for local health-care providers, that post-op outcomes be monitored and reported, and that short-term trips should be part of long-term partnerships [1,6].

Considering the important role that global health initiatives may play in addressing the unmet need for pediatric surgical care in SSA, it is important to assess the quality of previous international pediatric surgery partnerships in the region. This is essential for planning ethical and effective partnerships in the future. To date, there have only been two other literature reviews published on the subject. The first was a systematic literature review published in 2014 appraising literature published from 1990 to 2012 in three databases, which found 31 articles representing only 16 countries in SSA. From these, the authors defined four broad categories of pediatric surgery partnerships. None of the articles reviewed included data on training local health-care providers, and none measured any outcomes from the partnership activities [8]. The second was a general review article from 2016 about developing pediatric surgery in LMICs that did not follow the Preferred Reporting Items for Systematic Reviews and Meta-Analyses (PRISMA) guidelines, report methods, nor specify a date range for the articles reviewed [3].

The purpose of this study is to perform a scoping literature review of international pediatric surgery partnerships in SSA published from 2009 to 2019. We will categorize and critically assess all types of partnership activities in order to aid in the development of effective and sustainable global surgery efforts. This review will specifically address how many international pediatric surgery partnerships include education or training of local health-care providers, how many of these report educational outcomes, how many report post-op follow-up and patient outcomes, how many assess the attitudes of participants from SSA, how many include training of non-surgeon health-care providers, and how many report a funding source.

## Methods

### Search strategy and selection criteria

This study is a scoping literature review following the Preferred Reporting Items for Systematic Reviews and Meta-Analyses for Scoping Reviews (PRISMA-ScR) guidelines. Using the PubMed and Embase databases, we searched for articles published in English, French, and Portuguese from January 2009 – December 2019 that had a full text available. Searches were performed using 24 key words (Table 1) each combined with the additional search terms ‘surgery AND Africa AND (pediatric OR child)’. For articles published in French and Portuguese, Google Translate was used to translate abstracts and articles to English when an English version was not available.

**Table 1.** Keywords used in database searches.

Academic partnership	Mercy Ships
Capacity building	Mission
Charitable	Mission hospital
Collaboration	(Non-governmental organization OR NGO)
Global health	Outreach
Global surgery	Short-term surgical mission
Humanitarian	Task shifting
International elective	Telemedicine
International partnership	Twinning
International rotation	Volunteer
International surgeon	Volunteerism
(Medecins Sans Frontieres OR MSF)	

Inclusion criteria: article published in a peer-reviewed journal; article describes a specific surgery partnership between one country in SSA and another country either in SSA or a different continent (we used the Library of Congress Africana Collections list of countries in SSA [9]); article either specifically focuses on a pediatric patient population or includes demographic data showing patient age that includes patients <18 yo; and the article was a primary source for the surgery partnership described (not a review article).

The first author (AW) performed the initial data extraction, and the second author (CS) evaluated all articles along with AW to determine inclusion. The third author (BH) resolved any conflicts over article inclusion.

### Data analysis

All results of each search from both databases were imported into EndNote. We used EndNote software to identify and eliminate duplicate articles. Each full-text article included in the final review was read in-depth two to three times to determine if it included qualitative, descriptive information about training of health-care providers in SSA as part of the partnership activity, and if any educational outcomes were reported. Educational outcomes are defined as any measure of skill transfer, performance (such as exam scores), number of providers trained, or any other form of measured improvement reported after partnership activities took place.

Other data extracted included post-op patient outcomes, whether post-op follow-up was present, funding sources, education of non-surgeon providers including the type of provider, how long short-term surgical trips lasted and if they recurred in the same location, and if the article assessed the attitudes and perspectives of SSA partners. Descriptive analyses of the data collected were conducted in Excel.

### Funding information

There was no funding source for this study. The corresponding author had full access to all the data in the study and had final responsibility for the decision to submit for publication.

## Results

**Figure 1.**
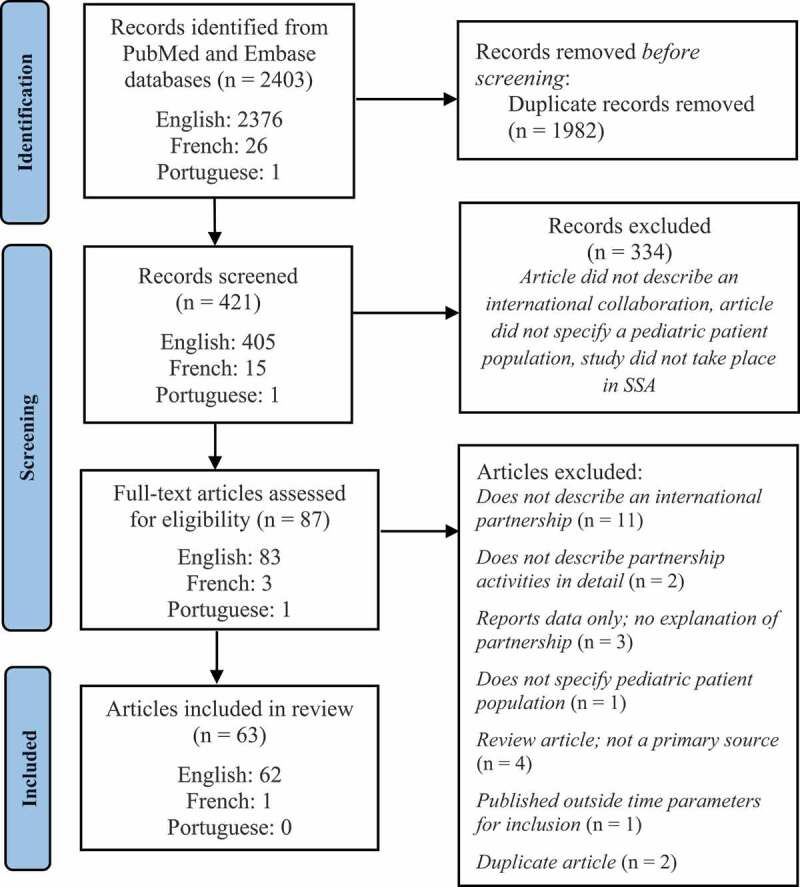
PRISMA 2020 flow diagram showing study selection and number of articles from each publication language.

Database searches identified a total of 2403 articles. After duplicates were removed, 421 articles were screened. In total, 87 full-text articles were assessed for eligibility, and 63 articles were included in the review. The database searches yielded significantly more English articles than French or Portuguese (Figure 1). In total, 18 categories of international pediatric surgery partnerships were identified (Table 2). In total, 34 articles were assigned to more than one category [10–43]. The most common category was short-term surgical trip [13,18,20,25–50] (29 articles, 46%) followed by NGO partnership [10,11,13,19–24,26–30,32–34,36–43,51–53] (28 articles, 44%). Within the short-term surgical trip category, trips ranged from three days to three months, with ten days and two weeks being the most common lengths. Twenty of the articles (71%) described short-term trips that recurred in the same location [12,21,25,27–32,34–37,39,41–44,46,47] (see Table A1).

**Table 2.** Categories of partnerships identified with definitions, results breakdown, and notes.

Category:	Definition:	Notes:
**Academic Partnership** **6 articles** -5 include education (83%) -3/5 report educational outcomes (60%) -Post-op follow-up and outcomes not applicable for this category -None assess attitudes of SSA participants	Partnership between two universities; a university and an academic teaching hospital; or an academic society and a university or academic teaching hospital. These partnerships are focused on pediatric surgical education for surgical trainees and practicing surgeons in SSA, along with supporting other activities such as research.	Two partnerships between an American university and a university in SSA [16,60]. Two partnerships between an American university and a teaching hospital in Kenya that has close ties to Kenyan universities [14,55]. One partnership was between an academic teaching hospital in the UK affiliated with Oxford University and a medical school in Tanzania. This included medical students from Tanzania completing surgery rotations in the UK [64]. Another partnership was between the American Society of Pediatric Otolaryngology and Addis Ababa University in Ethiopia [13].
**Fellow Exchange Program** **1 article** -Includes education -Reports educational outcomes -Post-op follow-up and outcomes not applicable -Assessed attitudes of both fellows about the program after the exchange	Pediatric surgery fellows from a country outside of SSA and pediatric surgery fellows from a country in SSA switch places and train at the other fellows’ institution for a short period of time (four to six weeks). The goal is for a mutually beneficial educational experience.	In an exchange between fellows in Canada and Kenya, Canadian fellows rated the exchange higher than Kenyan fellows in every category. Kenyan fellows had a harder time obtaining licensure and permission to operate and had more challenges completing the program (financial burden, impact on family, etc.) [68].
**Government Partnership** **7 articles** -6 include education (86%) -5/6 report educational outcomes (83%) -Follow-up and outcomes applicable for 6 articles (86%) -3/6 include post-op follow-up (50%) -5/6 include post-op outcomes (83%) -None assess attitudes of SSA participants	Partnership between the government of a country in SSA and an NGO, hospital, or university in a foreign country. Alternatively, a partnership between a foreign country’s government and an NGO, hospital, or university in SSA. The government provides resources with the aim of scaling up pediatric surgical care in the country in SSA.	Six partnerships between the government of a country in SSA and a hospital, government, or NGO in another country [15,17,22,25,31,32]. One partnership between the US government and a university in Mozambique [16]. Four government partnerships intended to establish pediatric cardiac surgical care in a country in SSA [15,17,31,32].
**International Hospital Partnership** **5 articles** -3 include education (60%) -2/3 report educational outcomes (67%) -Post-op follow-up and outcomes applicable for 5 articles (100%), 3 of these include post-op follow-up (60%) -5 include post-op outcomes (100%) -None assess attitudes of SSA participants	A partnership between a hospital in SSA and a hospital or NGO in another country with the aim of supporting pediatric surgical activity at the hospital in SSA. NGO’s provide financial support or coordinate follow-up & community health care for patients. Foreign hospitals send teams of pediatric surgical providers to the hospitals in SSA and provide opportunities for providers from SSA to receive pediatric surgical training at foreign institutions.	One partnership between an NGO and 7 pediatric surgical centers in SSA. The NGO provides financial support, needs assessments, and quality improvement measures [65]. Three partnerships between a hospital in SSA and a hospital in another country. Healthcare providers from the other country visit the SSA hospital regularly to provide pediatric surgical care and education to local providers [15,27,31]. In one partnership surgical providers from SSA travel to a partner hospital in another country to receive extended training in pediatric surgery [27].
**International Patient Transfer** **7 articles** -One includes education (17%) -Post-op follow-up and outcomes applicable for 6 articles (100%) -5 include post-op follow-up (71%) -5 include post-op outcomes (71%) -None assess attitudes of SSA participants	Pediatric patients from a country in SSA are transferred to a foreign country for complete surgical care. This is either as the only means for local patients to receive any surgical care, or for complex patients requiring specialty surgical care that is not available in the patients’ country of origin.	Four international patient transfers for pediatric cardiac surgery as patients’ only means for obtaining cardiac surgery of any kind [17–20]. Three international patient transfers as a part of larger partnership activities with an institution in SSA, where only the most complex patients that cannot be managed by providers in SSA are transferred to foreign countries for care [21,27,43].
**International Surgery Rotation** **2 articles** -2 include education (100%) -None report educational outcomes -Post-op follow-up and outcomes not applicable for this category -None assess attitudes of SSA partners	Medical students, general surgery residents, or pediatric surgery fellows from a country outside SSA complete clinical rotations at a partner institution in SSA and can receive elective credit for the rotation.	One article mentions that the American trainees (surgery residents) are expected to be both learners and teachers at the partner institution in SSA [14]. One article includes medical students from the partner institution in the UK traveling to Kenya, but Kenyan medical students did not go to the UK institution in return [35].
**International Surgical Society/Symposium** **4 articles** -4 include education (100%) -None report educational outcomes -Post-op follow-up and outcomes not applicable for this category -None assess attitudes of participants	An international society of pediatric surgeons from multiple countries around the world (including SSA) that organizes regular meetings and activities [56,66,67], or a symposium meeting in which pediatric surgeons from around the world (including SSA) gather without being organized into a formal society [61].	International Surgical Societies include regular symposium meetings and on-going activities such as a society peer-reviewed journal and pediatric surgery database creation and maintenance for use by members [66]. Symposiums and society meetings include lectures and workshops on specific pediatric surgery topics as well as poster and podium presentations.
**Long-term Surgical Outreach** **2 articles** -None include education -Post-op follow-up and outcomes applicable for 2 articles (100%) -1 includes post-op follow-up (50%) -1 includes post-op outcomes (50%) -None assess attitudes of host country participants	Surgical activity performed by a partner country organization in a host country in SSA for a continuous period longer than one year.	Organizations that ran & funded the long-term outreaches: 1. International Committee of the Red Cross [23] 2. Médecins Sans Frontières [24]
**Long-term Surgical Volunteers** **3 articles** -1 includes education (33%) -Does not report educational outcomes -Post-op follow-up and outcomes applicable for 3 articles (100%) -None include post-op follow-up or patient outcomes -None assess the attitudes of host country participants	A single expatriate pediatric surgeon practices surgery in a country in SSA for a continuous period longer than one year.	An American pediatric neurosurgeon helped found CURE Children’s Hospital of Uganda and practiced there for six years. He trained Ugandan surgeons and medical officers and the hospital is now fully staffed by Ugandans [62]. An American pediatric surgeon practiced for two-and-a-half years at a government hospital in The Gambia [63]. One expatriate cardiothoracic surgeon was contracted by the Kenyan government to develop a cardiac surgery program in Kenya [25].
**Military Surgical Mission** **1 article** -Does not include education -Post-op follow-up and outcomes applicable -Does not include post-op follow-up or outcomes -Does not assess the attitudes of the local population	Surgery outreach performed by foreign military personnel in a country in SSA for a defined period. Must include operations on local civilians in addition to war-related surgeries [72].	22-month mission. At least half of the mission took place during peacetime. 63% of all surgeries performed were on Central African Republic citizens, and most of these were elective.
**NGO Partnership** **28 articles** -13 include education (46%) -7/13 report educational outcomes (54%) -Follow-up and outcomes applicable for 22 articles (79%) -9/22 articles include post-op follow-up (41%) -15/22 include post-op outcomes (68%) -2/28 assess the attitudes of SSA participants (7%)	Partnership between an NGO and a hospital, healthcare system, NGO, or university. The NGO may be based in a country in SSA or a country outside of SSA.	In 3 articles, the role of the NGO partner was funding only [19–21]. Activities of NGO partners include organizing short-term surgical trips, recruiting pediatric surgeons to go on surgical trips, and long-term partnerships of continuous support to healthcare institutions in SSA.
**Residency/Fellowship Training Program** **2 articles** -2 include education (100%) -2 report educational outcomes (100%) -Post-op follow-up & outcomes not applicable -None assess attitudes of participants	A general surgery residency program that includes training in pediatric surgery [54] or a pediatric surgery fellowship program in SSA developed in collaboration with surgical faculty and/or organizations in a country outside SSA [14].	One residency program developed by Vanderbilt University (US) and University of Nairobi (Kenya) [14]. General surgery residencies and pediatric surgery fellowships developed by the Pan African Academy of Christian Surgeons and surgical faculty from the US, with accreditation through Loma Linda University (US) and the West African College of Surgeons [54].
**Short-term Rotating Teams** **2 articles** -2 include education (100%) -1 reports educational outcomes (50%)-Follow-up & outcomes applicable in 1 article -This article reports post-op follow-up and outcomes -None assess attitudes of host country participants	Visiting faculty and healthcare personnel go to a single facility in SSA for a short period of time (one or more weeks) on a rotational schedule so that visiting team members are present continuously throughout the year. This can either be for education (visiting surgical faculty) or to have a continuous presence of foreign healthcare providers present to perform surgical care.	ENT faculty from America visit Addis Ababa University in Ethiopia for one week every month of the year to provide a continuous chain of educators for Ethiopian ENT surgery trainees [13]. 13-member multidisciplinary teams from Portugal visit a hospital in Luanda, Angola on a rotational basis throughout the year so that a multidisciplinary team is always present to provide pediatric cardiac surgery to Angolan patients and education to local providers [15].
**Short-term Surgical Trip** **29 articles** -15 include education (52%) -8/14 report educational outcomes (57%) -Follow-up and outcomes applicable for 28 articles (96%) -12/28 include post-op follow-up (43%) -18/28 include post-op outcomes (64%) -2/29 assess attitudes of SSA participants (7%)	Surgeons and healthcare personnel from a country outside of SSA travel to a country in SSA to provide surgery for local patients and/or education to local healthcare providers for relatively short period of time (less than one year).	The length of short-term trips ranged from 3 days to 3 months. The most common length of time was 2 weeks (11 articles, 39%) [21,26–28,36,37,41,45,46,48]. 9 articles did not specify the length of the trip (14%) [12,18,29,34,39,41,44,47,49]. 20 articles (69%) describe short-term trips that are recurring (return to the same location more than once) either annually, biannually, or at an unspecified periodicity [12,21,26–32,34–37,39,41,44,46,47].
**Surgical Camp** **3 articles** -3 includes education (100%) -2/3 report educational outcomes (67%) -Post-op follow-up and outcomes applicable for 3 articles -1 includes post-op follow-up (33%) -2 include post-op outcomes (67%) -None assess attitudes of host country participants	A team from the partner country and a team from the host country go on a joint surgical outreach to a defined area of need within the host country to perform operations over a short period of time (one to two weeks). May or may not focus on a single surgical condition or procedure.	Using joint teams with members from both countries allows for maximal knowledge & skill sharing [58]. Focusing on one procedure allowed host country trainees to become proficient in the operation by the end of the camp. It may take multiple camps for proficiency in complex operations [57]. One camp operated on patients on the waitlist for neurosurgery in the local healthcare system [59].
**Surgical Ships** **2 articles** -None include education -Post-op follow-up and patient outcomes applicable for 2 articles -1 includes post-op follow-up (50%) -2 include post-op outcomes (100%)	Fully equipped hospital ship visits countries in SSA and provides surgery to the local population [70,71].	Allows foreign providers to bring all the infrastructure needed for surgical care with them. Limited opportunities for educating and involving local providers. Not a sustainable model.
**Surgical Simulation Workshop** **2 articles** -2 include education (100%) -1 reports educational outcomes (50%) -Post-op follow-up and outcomes not applicable for this category -1 assessed attitudes of host country participants (50%)	A training experience that teaches providers from SSA how to perform a specific procedure using dummies and/or simulation software. Workshops may include didactic lectures on surgical content.	One workshop focused on the neuroendoscopic ventriculostomy procedure for hydrocephalus using a portable neuroendoscopy system [10]. One workshop focused on cleft lip and palate repairs using high-fidelity cleft lip and palate simulators [11].
**Telemedicine** **3 articles** -3 include education (100%) -One reports educational outcomes (33%) -Post-op follow-up and outcomes applicable for 3 articles -1 includes post-op follow-up (33%) -1 includes post-op outcomes (33%)	Collaboration between surgeons in SSA and in a foreign country by transmission of patient information through email with consultations between surgeons over the phone, email, or video, or live, joint assessments of surgical patients via video conference.	In two articles, telemedicine was used to screen patients pre-operatively and assess them post-operatively before and after short-term surgical trips [12,43]. In another article, telemedicine was used for general surgeons in SSA to consult pediatric orthopedic surgeons for management of complex pediatric orthopedic cases [69].

**Figure 2.**
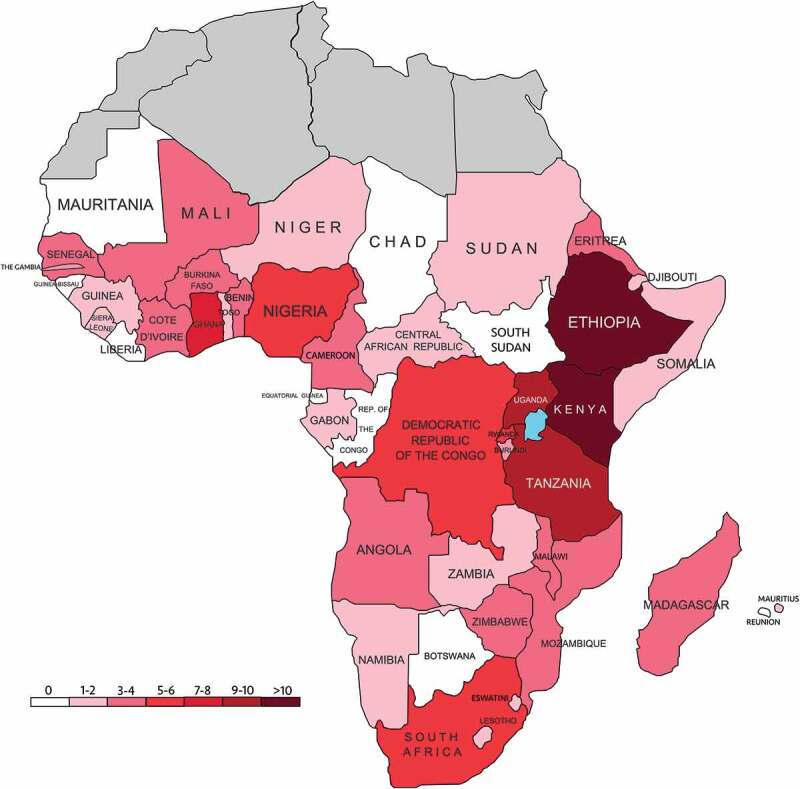
Heat map indicating the number of articles in which each country in SSA is mentioned.

Thirty-seven countries in SSA were represented (Figure 2). In this review, the term ‘host country’ refers to a country in SSA where partnership activities take place, and ‘partner country’ refers to another country (either in SSA or another region) that travels to the host country for partnership activities. Fifteen partner countries were identified (Table 3). In two articles, a partner country was another country in SSA (South Africa [17] and Kenya [10]). In three articles, a partner country was another LMIC outside the SSA region (Brazil [16], Egypt [50], and India [47]). The most common host countries were Kenya (12 articles [11,14,25,35,38,46,49,51,52,54–56]:) and Ethiopia (11 articles [10,13,25,28–30,40,42,51,52,57]:). The most common partner countries were the USA (US) (27 articles [10,11,13,14,16,22,25,30,32–34,37,39,45–48,51,52,54,55,58–63]:) and the UK (11 articles [25,28,35,36,40,42,47,56,61,64,65]:).

**Table 3.** Partner countries and how many articles in which each appears.

USA	27	Switzerland	2
UK	11	Brazil	1
France	8	Egypt	1
Canada	5	India	1
Spain	5	Ireland	1
Italy	4	Kenya	1
Germany	3	South Africa	1
Portugal	2		

**Figure 3.**
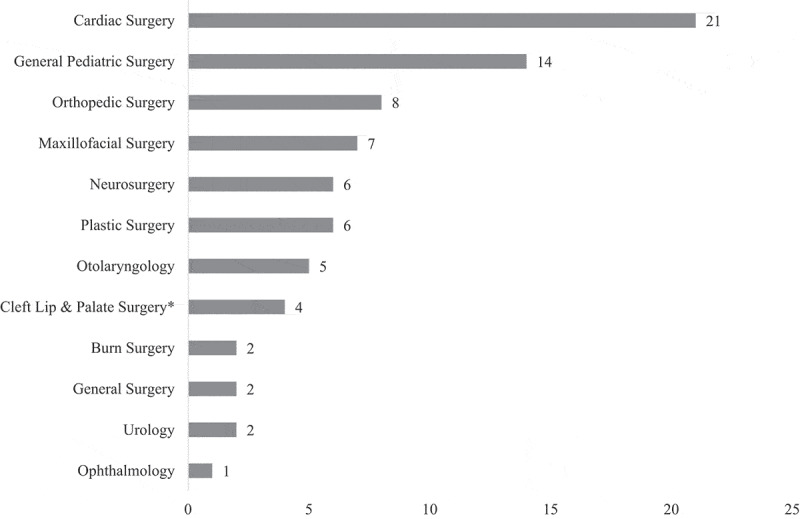
Types of surgery and the number of articles in which each appears. *Cleft lip and palate surgery can be performed by multiple surgical specialists, so when the type of surgeon was not defined these were included in a cleft lip and palate category.

Twelve surgical specialties were represented, with cardiac surgery (21 articles, 34%) [14,15,17–20,22,25,29,32,37,39–42,47,48,53,61,66,67] and general pediatric surgery (14 articles, 23%) [14,16,27,31,45,50,54,56,58,60,63–65,68] being the most common (Figure 3).

Thirty-six articles (57%) included education or training of host country health-care providers as part of the partnership activities [10–17,22,25–27,29,31,33–36,38,42–45,54,56–62,64,66–69]. Of these, 19 articles (53%) reported an outcome measure for the educational activities [10,14–16,22,25,31,33,36,38,42,43,45,54,57,59,62,64,68] (for a list of educational outcomes reported, see Table A2). In the short-term surgical trip category, 15 of 29 articles (52%) included education, followed by NGO partnership (13/28, 46%), long-term surgical volunteers (1/3, 33%), and international patient transfer (1/6, 17%). Five articles (8%) included training general surgeons in pediatric surgical techniques [31,33,34,60,64]. In total, 17 articles (27%) included education or training for non-surgeon, peri-op health-care providers, such as nurses or anesthetists [10,11,15,16,22,26,28,31,33,36,38,42,58–60,62,64] (see Table A3 for the types of peri-op providers trained).

In 15 articles, partnership activities focused on education only or did not involve direct operation on host country patients. For these articles, post-op follow-up and patient outcomes were deemed not applicable [10,11,13,14,16,45,54–56,60,61,64,66–68]. In the remaining 48 articles, 21 (45%) included post-op follow-up care [12,15,17,18,20–23,27,30,32,33,35,41,42,44,49,53,58,70,71] and 31 (66%) included post-op patient outcomes [15,17,18,20–23,27,28,30–36,39,41,42,44,46,49,51–53,58,59,65,69–71]. The most reported outcomes were mortality (22/31 articles, 71%) and post-op complications (17/31 articles, 55%) (for a list of outcomes reported, see Table A4).

Thirty-three articles (52%) identified a funding source for the partnership activities described [10,15–17,19–25,27–30,33,38–40,42,45,52,53,55,56,58,60,62,65,68,70–72]. The most common funding source was an NGO (27/33 articles, 81%) (for a list of funding sources, see Table A5). Four articles (6%) included an assessment of the attitudes and perspectives of host country participants about the partnership [11,29,45,68] (for a list of factors assessed, see Table A6). In two articles, no outcome measures were found (no education, follow-up, patient outcomes, or funding source) [48,63].

## Discussion

While the articles included in this review do not cover all international partnerships in pediatric surgery that have taken place in SSA, they represent a snapshot of partnership work in the past decade. An essential consideration in evaluating global health partnerships is whether they include education of local health-care providers. The Lancet Global Surgery 2030 report recommended that all international surgery partnerships contain a training component outside of the acute crisis setting [1]. Just over half of the articles in this review included education of host country providers. This is an improvement compared to a previous systematic review done on this subject in which no articles contained data on training of host country providers outside of four programs that sent surgeons from SSA to train in the US [8]. However, much work needs to be done to ensure that all partnerships contain education in the future.

Of note, only half of the articles that did include education provided measures of educational outcomes. The most frequently reported outcome, reported in only seven of the articles, was the ability of host country surgeons and trainees to perform surgeries independently [10,12,15,25,31,33,59]. To ensure that training during international partnerships is effective, partnerships need to design and report educational outcome measures. The ability of host country providers to operate independently is a good benchmark for all partnerships to strive for.

Short-term surgical trips were the most common category of partnerships encountered. This is not surprising, as short-term trips have dominated global surgery efforts for years [3,7]. There are many potential pitfalls of the short-term surgical trip. They are often not sustainable or have limited impact on the overall burden of disease for a community [7], may disrupt and overburden the local healthcare system [3], and may leave patients and their local physicians without adequate provisions for follow-up care [3,73]. To ensure sustainability, short-term trips should include a training component. However, only half of the short-term surgical trips in this review included education [12,25,26,28,29,31,33–36,38,42,44,45]. Short-term trips are more sustainable when they recur in the same location more than once as part of a long-term partnership [6], and more than two-thirds (71%) of the short-term surgical trips in this review did recur. When planning future short-term surgical trips, aiming for a long-term investment in one location is vital.

Post-op follow-up is often an essential component of surgical care and is of particular importance in short-term trips, when surgeons may depart soon after operations are performed. Less than half of the short-term surgical trips included post-op follow-up care for patients when applicable [12,18,21,23,30,32,33,35,41,42,44,49]. Among all categories, less than half (45%) of articles reported post-op follow-up when applicable. This represents a serious shortcoming that future pediatric surgery partnerships in SSA must address.

One strategy that could aid in the provision of follow-up after surgical partnerships is the use of telemedicine. Only three articles in the review utilized this platform. The two used telemedicine to jointly pre-select patients with host-country partners for surgery on short-term trips [12,43], and one of these also used telemedicine for follow-up after the trips [12]. This is a cost-effective way to ensure adequate follow-up care and to build mutual partnering relationships with providers in SSA. The third article utilized telemedicine as a way for general surgeons to consult pediatric specialist surgeons in a partner country without the need for either partner to travel [69]. This could be an effective way for surgeons in partner countries to support pediatric surgery development in SSA at minimal cost and while maintaining the autonomy of the host country provider.

Closely related to follow-up care are post-op surgical outcomes. Data suggests that operations performed through humanitarian platforms may have higher complication rates and poorer clinical outcomes than those performed in high-income-country (HIC) hospitals [73]. There can also be a lack of accountability for poor outcomes when visiting surgeons leave the host country [3,37]. For these reasons, it is imperative that all international partnerships report patient outcomes when applicable. Of the 48 articles in which patient outcomes were applicable (partnership activities involved operating on host country patients), the majority (66%) reported them. However, this still leaves a large proportion of partnerships that did not report patient outcomes, highlighting another area requiring improvement in future partnerships. In addition to mortality and complications, guidelines suggest that quality-of-life outcomes should also be reported for global surgery initiatives [6]. Only seven articles included quality of life outcomes (functionality [23,27,35,44,69], ability to attend school or work [70], feelings of shame and acceptability in society [71], and being teased by others [70]).

An important consideration when designing truly sustainable surgical initiatives is the availability of the necessary peri- and post-op staff including nurses, anesthetists, technicians, and so on. Only a minority of articles (17%) described surgical initiatives that included specific training of non-surgeon peri-op providers [10,11,15,16,22,26,28,31,33,36,38,42,58–60,62,64], indicating that this may be an important focus of future capacity-building efforts. In addition, many surgeries may also require intensive care unit (ICU)-level care post-op. Two articles highlighted the challenges of caring for patients postoperatively with inadequate ICU infrastructure. In one, a local, general pediatrician in Ghana was left to manage open-heart surgery patients post-op who required ICU-level specialist care that both he and the hospital were not equipped to provide [37]. In another, a visiting neurosurgeon in Kenya found that the surgery he provided was not beneficial if patients could not receive the necessary level of peri-op care. He subsequently spent most of his time training local nurses and physicians in neuro-ICU patient management [38]. Ensuring that local hospitals have adequate peri-op staff and post-op infrastructure for the surgeries performed is an essential consideration for international surgery partnerships.

Considering the length of time it takes to become a fully certified pediatric surgeon, one way to increase pediatric surgery capacity in SSA is to train general surgeons in high-yield pediatric surgery techniques. This may be the most efficient way to ensure more equitable access to pediatric surgery for patients in the region. Only 5 articles in the review included the training of general surgeons in pediatric surgery procedures [31,33,34,60,64]. Future partnerships should consider incorporating this strategy into their plans.

When assessing the efficacy of international pediatric surgery partnerships, it is important to consider the attitudes and perspectives of host country participants. However, only four articles included such an assessment [11,29,45,68]. In one, Canadian and Kenyan pediatric surgery fellows who participated in an exchange program filled out detailed surveys after the program. The results revealed that Kenyan fellows experienced more challenges in the exchange program and rated the exchange lower in every category compared to the Canadian fellows. Lower ratings were attributable to the fact that it was more difficult for Kenyan fellows to get full licensure to perform surgery in Canada, so some had to only observe surgeries for the whole program. All Canadian fellows were able to participate fully in surgeries in Kenya [68].

A survey of Ethiopian surgeons after a short-term surgical trip showed that they felt overseas missions should enter into an agreement with the Federal Ministry of Health before arriving and should limit the number of people on visiting teams so that more Ethiopian providers could participate. They also commented that visiting surgeons should plan to operate on many patients with a similar diagnosis so that Ethiopian surgeons could gain the repetition needed to master a single type of procedure [29].

The insights gained from these assessments of local participants’ perspectives show critical needs for improvement in future partnership activities. Assessing the viewpoints of SSA partners could help identify blind spots in the partnership by pointing out problems and different perspectives between the visitors and hosts. Addressing these areas could lessen any unintended harm from the visiting surgical team, improve outcomes, and secondarily strengthen the partnership. Thus, moving forward, every partnership should evaluate the perspectives of their SSA partners.

Finally, to promote maximal transparency, publications on international pediatric surgery partnerships should disclose the funding source(s) for the partnership and/or how funding was raised. Just over half (52%) of the articles in this review identified a funding source, but more partnerships need to do so in the future.

The limitations of this study include that we only evaluated two databases and did not conduct a search of the grey literature. Directions for future study include investigating initiatives to train anesthesia and peri-op nursing providers in SSA, as well as specific initiatives to train pediatricians in SSA to manage peri-op care, and reviewing the literature on pediatric surgery partnerships in southeast Asia, another region with great unmet need for surgical care [1].

## Conclusions

In conclusion, international partnerships can play an important role in helping to scale up the critically low pediatric surgery capacity in SSA. We recommend that all partnerships include an element of education for host country surgeons and that they measure and report educational outcomes. Education should also extend to peri-op staff and general surgeons. All partnerships should ensure adequate follow-up care for patients after visiting surgeons depart, and all should report post-op patient outcomes. The perspectives of participants from SSA should be regularly evaluated. Publications about partnership activities should disclose funding sources. Currently, too few partnerships are meeting these recommendations, pointing to the need for improvement when planning future initiatives.

All the points we have raised stress the need to form true partnerships with leaders from the SSA sites. They must be involved and have the leading voice at every level of the partnership, from building the necessary infrastructure, training appropriate personnel, selecting the appropriate patients and procedures, ensuring needed follow-up, and assessing outcomes.

## Paper context

Only two prior review articles have been published on this topic to date. One was a systematic review of articles from 1990 to 2012. Another was a general review about pediatric surgery in LMICs which did not report any methods. This study focuses on more recent literature to provide an updated review following PRISMA guidelines. It provides a more detailed categorization of pediatric surgery partnerships and previously unreported data about education, post-operative follow-up, outcomes, and funding sources.

## Appendix: additional tables

**Table A1:** Length of time of short-term surgical trips and the number of articles in which each occurs. Number of short-term trips that recur in the same location and the periodicity with which they recur.

Short-term Surgical Trips	
Length of trip:	Trips that recur:
Ten days – 11	Total – 19
Two weeks – 11	Recur annually – 8
Not specified – 9	Recur biannually – 2
One week – 4	Periodicity not specified – 9
Three to four days – 3	
Three weeks – 1	
Two months – 1	
Three months −1

**Table A2:** Educational outcomes reported and the number of articles in which each is reported. (SSA = sub-Saharan Africa).

Ability of local surgeons and/or surgical trainees to perform operations independently	7
Improvement in peri-operative care by nursing staff	4
Number of surgical trainees that are now practicing pediatric surgeons in SSA	4
Number of non-surgeon providers trained in peri-operative care	2
Number of publications by SSA trainees & physicians	2
Number of trainees now serving as academic faculty in SSA	2
Average number of cases performed by surgical fellows during partnership	1
Board exam performance	1
Development of a structured curriculum for surgical residents	1
Hospital in SSA now staffed entirely by local providers	1
Improved English language proficiency of SSA trainees	1
Improved time efficiency of surgeries	1
Increased confidence & autonomy of local providers	1
Increase in number of local instructors & mentors	1
Increased staff satisfaction & retention	1
Number of first-author publications by SSA trainees & physicians	1
Number of presentations at international meetings by SSA trainees & physicians	1
Number of surgeons & residents trained in a specific procedure	1
Number of surgeries performed as first surgeon by local trainees	1
Number of trainees going on to pediatric surgery fellowships	1
Number of trainees who have received fellowships from international pediatric surgery associations	1
Pre- and post-test scores	1

**Table A3:** Non-surgeon health-care providers trained during partnership activities and the number of articles in which each is mentioned.

Nurses	13
Clinical/medical officers	5
Anesthesiologists/anesthetists	3
Cardiologists	2
Hemodynamic technicians	1
Paramedics	1
Post-op recovery staff	1
Speech pathologists	1
Surgical technicians	1

**Table A4:** Post-operative outcomes reported and the number of articles in which each was reported (DALY = disability adjusted life years, ICU = intensive care unit).

Mortality	22
Post-op complications	17
Functionality*	6
Requiring multiple operations or redo surgery	6
Symptom improvement	4
DALYs	3
Patient satisfaction	3
Cure or improvement in condition	2
Morbidity	2
Ability to attend school or work	1
Being teased by others	1
Condition worsening or failing to improve	1
Feelings of shame & acceptability	1
Length of post-op ICU stay	1
Median length of hospital stay	1
Patient’s expectations met or not met	1
Patient’s perception of care	1

*Functionality outcomes included assessments of disability, ambulation, speech, and hearing.

**Table A5:** Sources of funding identified for partnership activities and the number of articles in which each is reported.

NGO	23
Government	7
Private donations	3
Academic institution	2
Donated supplies	2
Endowment	2
Grant funding	2
Volunteer host families that house patients for free	2
Funding mutually raised by both partners	1
Pharmaceutical firms	1
Scholarship	1
State company	1

**Table A6:** The attitudes and perceptions of host country participants about the partnership activities and the number of articles in which each measure was reported.

Would recommend partnership activity to a colleague	2
Gained valuable clinical knowledge from partnership activity	1
Gained valuable technical skills from partnership activity	1
Increase in trainee confidence after partnership activity	1
Partnership activity met expectations	1
Perceived challenges to trainees’ participation in the partnership	1
Surgical roles of local trainees & surgeons during partnership activity (1^st^ assist, 2^nd^ assist, primary surgeon, observer)	1
Would participate in partnership activity again	1
Partnership activity was worth the time & effort involved	1
